# Phenolic and Volatile Composition of a Dry Spearmint (*Mentha spicata* L.) Extract

**DOI:** 10.3390/molecules21081007

**Published:** 2016-08-03

**Authors:** Martina Cirlini, Pedro Mena, Michele Tassotti, Kelli A. Herrlinger, Kristin M. Nieman, Chiara Dall’Asta, Daniele Del Rio

**Affiliations:** 1 Department of Food Science, University of Parma, Parma 43125, Italy; martina.cirlini@unipr.it (M.C.); michele.tassotti@studenti.unipr.it (M.T.); chiara.dallasta@unipr.it (C.D.); 2 Kemin Foods, L.C., 2100 Maury Street, Des Moines, IA 50317, USA; kelli.herrlinger@kemin.com (K.A.H.); kristin.nieman@kemin.com (K.M.N.); daniele.delrio@unipr.it (D.D.R.)

**Keywords:** spearmint, phenolic composition, volatile fraction, phytochemical characterization, UHPLC-ESI-MS^n^, HS-SPME/GC-MS

## Abstract

The present paper reports a complete mass spectrometric characterization of both the phenolic and volatile fractions of a dried spearmint extract. Phenolic compounds were analysed by ultra-high performance liquid chromatography-electrospray ionization-mass spectrometry (UHPLC-ESI-MS^n^) and a total of 66 compounds were tentatively identified, being the widest phenolic characterisation of spearmint to date. The analysis suggests that the extract is composed of rosmarinic acid and its derivatives (230.5 ± 13.5 mg/g) with smaller amounts of salvianolic acids, caffeoylquinic acids, hydroxybenzoic acids, hydroxycinnamic acids, flavones, and flavanones. Head space solid-phase microextraction (HS-SPME) coupled with gas chromatography-mass spectrometry (GC-MS) technique, that was applied to characterize the volatile fraction of spearmint, identified molecules belonging to different chemical classes, such as *p*-cymene, isopiperitone, and piperitone, dihydroedulan II, menthone, *p*-cymen-8-ol, and β-linalool. This comprehensive phytochemical analysis can be useful to test the authenticity of this product rich in rosmarinic acid and other phenolics, and when assessing its biological properties. It may also be applied to other plant-derived food extracts and beverages containing a broad range of phytochemical compounds.

## 1. Introduction

Among the family of Lamiaceae (Labiatae), mint represents one of the most popular and cultivated officinal and aromatic plants [1]. The cultivation of mint is principally in temperate regions of Europe and Asia, but also in South Africa, Australia, and the United States.

Spearmint (*Mentha spicata* L.) is an aromatic plant that can be used fresh or as dried leaves or powder, as a seasoning and flavouring herb, or traditionally as an herbal tea. It is commonly used in traditional medicines as a remedy for gastrointestinal and respiratory problems. In addition, spearmint essential oil has economic relevance due to its use in perfumery, confectionary, and pharmaceutical preparations. Besides its flavouring properties, spearmint is also widely used as an antimicrobial agent and as a preservative in food, mainly on account of the phenolic and terpenoid content [2].

The volatile (non-polar) profile of traditional cultivars of spearmint essential oils is mainly constituted by carvone (22%–73%) and limonene (8%–31%), with smaller quantities of 1,8-cineole (4%–7%), menthone (1%–5%), menthol, eucalyptol, and other minor compounds. The profile varies based on plant variety, growth, climate conditions, and harvest time [3,4,5]. The antimicrobial activity of these spearmint essential oil components has been widely described in the literature. Volatile molecules are indeed produced by the plant, serving as a defence mechanism upon predator attack (i.e., pathogens and insects) [5].

Polar extracts of spearmint leaves are, on the contrary, characterised mainly by a high content of phenolic compounds such as rosmarinic acid, luteolin, and apigenin derivatives [6,7]. Some of these components have been shown to have antioxidant properties; therefore, *Mentha spicata* could also be considered an antioxidant source [7]. In fact, spearmint and spearmint extracts are often used as preservative agents to delay the oxidative degradation that occurs in food during processing or over time with storage [1]. More intriguingly, the anti-inflammatory properties of spearmint extracts rich in phenolic compounds have been demonstrated in vivo in rats [8].

Aqueous extracts from typical commercially grown spearmint lines reportedly contain 0%–6% rosmarinic acid on a dry weight basis [9,10]. However, based on the reported benefits of rosmarinic acid, spearmint lines were developed through selective-breeding techniques to contain higher levels of phenolic compounds such as rosmarinic acid [11]. Therefore, this study aimed to comprehensively characterise the phytochemical profile of a dried aqueous extract from these proprietary spearmint lines. The phenolic composition was fully examined by means of UHPLC-ESI-MS^n^, while the composition of the volatile fraction was investigated using head space solid-phase microextraction (HS-SPME)/GC-MS technique.

## 2. Results and Discussion

### 2.1. Characterization of the Phenolic Profile

**Figure 1:**
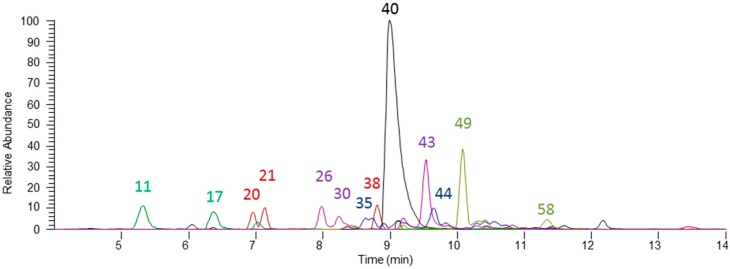
Main spearmint phenolics identified in the extract. Peak numbers are based on Table 1.

The phenolic fraction of spearmint was fully characterised by means of UHPLC-ESI-MS operating in two complementary conditions. The comprehensive evaluation of the sample allowed for the tentative identification of a total of 66 compounds (Table 1), the widest phenolic characterisation of spearmint to date. More than 200 mass spectrum outputs were analysed for each analytical replicate and experimental condition. Among the classes of identified (poly)phenolic compounds in spearmint, rosmarinic acid derivatives and salvianolic acids were the most prevalent (Figure 1). Different flavones, flavanones, flavonols, phenolic acids, and lignans were also detected. In addition, other phytochemicals, such as organic acids were found.

The retention times and mass spectrum data, reported as peak assignments for the identified phytochemicals, are included in Table 1. Twelve of the 66 identified compounds were identified and quantified by comparison with reference standards. The remaining 54 compounds were tentatively identified based on the interpretation of their mass spectral behaviour obtained from MS^2^ and MS^3^ experiments, and by comparing with data from the literature.

The 54 compounds tentatively identified according to their mass spectral behaviour were quantified by comparison with reference compounds selected based on structural similarity and considering that the functional groups may strongly affect their ionisation properties (i.e., salvianolic acid J was quantified as salvianolic acid A, salvianolic acid E as salvianolic acid B, danshensu and its derivatives as caffeic acid, etc.). Accordingly, in this case, data reported in Table 2 must be considered as semi-quantification. Nevertheless, some compounds responded to the electro-spray ionisation in a unique manner relative to the reference standards used or did not reach the limit of quantification (LOQ) of the corresponding reference compound; therefore, they were not quantified to avoid miscalculation of the phenolic content of the spearmint extract.

The total amount of phenolic compounds of the evaluated spearmint extract calculated on the basis of UHPLC-ESI-MS^n^ data was 262.97 ± 15.90 mg/g, which was in agreement with Dorman et al. [7], who reported a total phenolic content for *Mentha spicata* L. (spearmint) extract of 214 mg/g, expressed as gallic acid equivalents. More specifically, the sum of rosmarinic acid and other rosmarinic acid derivatives (such as sagerinic acid) in this extract was about the 88% (230.50 ± 13.50 mg/g) of the total amount of detected phenolics, followed by the sum of salvianolic acids (5.6% of total phenolics, 14.70 ± 1.19 mg/g) and caffeoylquinic acids (1.2% of total phenolics, 3.06 ± 0.27 mg/g). Hydroxycinnamic acids, including caftaric acid (an ester of caffeic and tartaric acids), represented about 1.1% of total phenolics (3.00 ± 0.36 mg/g). All of the other detected phenolic groups, such as flavonols, flavanones, flavones, hydroxybenzoic acids, and hydroxyphenylpropanoic acids represented approximately 1% of the total amount of phenolic compounds (0.01 to 0.99 mg/g).

Among the detected compounds, rosmarinic acid, a caffeic acid dimer, was identified by comparing the mass spectra obtained for the sample with those registered for a rosmarinic acid standard solution. This compound occurred at the highest concentration (173.76 ± 11.52 mg/g) and is approximately four-fold higher than the 4.6 mg/g reported for other water extracted spearmint lines [7]. Differences in the amount of rosmarinic acid of this extract with respect to other spearmint extracts are likely due to the selective-breeding techniques used for its production. However, rosmarinic acid concentrations could vary due to seasonal growth or extraction procedures. Rosmarinic acid is known to exert anti-inflammatory activities mainly due to its ability to inhibit lipoxygenases and cyclooxygenases, but it has also been shown to have anti-acetylcholinesterase, antioxidant, and antibacterial capabilities [28,29,30]. Furthermore, it was possible to observe the presence of several rosmarinic acid derivatives. In particular, significant amounts of sagerinic acid (8.93 ± 1.10 mg/g) and an isomer of sagerinic acid (peak 41; 40.05 ± 2.20 mg/g) were found. This is consistent with results obtained from analysis of lemon balm extracts [25], but have not been reported in the literature in water-extracted spearmint to date.

Other polar compounds in the spearmint extract included additional caffeic acid derivatives, such as salvianolic acids. Among this group of molecules, salvianolic acid A was the most abundant (7.79 ± 0.52 mg/g), followed by salvianolic acid B (1.35 ± 0.16 mg/g). Both were identified by means of reference compounds and served to identify their respective derivatives and isomers. Salvianolic acid D and F (dimers of caffeic acids), salvianolic acid J (a trimer of caffeic acid), and salvianolic acid E (a tetramer of caffeic acid), were all recognised by comparing the obtained fragmentations with those observed following analysis of extracts from *Salvia miltiorrhiza* roots [14]. All of these compounds displayed the characteristic mass spectra of salvianolic acids: neutral losses of one caffeic acid molecule (*m*/*z* 180) and a danshensu unit (*m*/*z* 198). Salvianolic acids have been reported in other members of the Lamaciae family although inconsistent between species. Within the *Mentha* species, data on salvianolic acid concentrations within water extracts is limited, with concentrations of less than 1% observed in some instances and slightly lower than the currently evaluated extract [6]. Danshensu (dyhydroxyphenyllactic acid), another caffeic acid derivative, as well as other danshensu-like compounds (peaks 53, 54, and 55) were identified on the basis of its molecular ion [M − H]^−^ (*m*/*z* 197) and its MS^2^ and MS^3^ fragments (*m*/*z* 179, 153 and 135) [14]. Moreover, a considerable amount of lithospermic acid (3.81 ± 0.26 mg/g), a caffeate trimer, was identified using a reference standard.

The presence of different hydroxycinnamic acids was observed in the first part of the chromatogram. This category was mainly represented by caftaric acid (2.18 ± 0.30 mg/g), followed by caffeic acid (0.71 ± 0.06 mg/g) and other minor components, such as dicaffeic acid and coumaric acid. The phenolic profile contained some compounds in the caffeoylquinic acid family, identified by their respective commercial standards (chlorogenic acid and neochlorogenic acid) or its characteristic fragmentation patterns (feruloylquinic acid). Small amounts of hydroxybenzoic acids were detected (0.57 ± 0.07 mg/g) and the presence of salicylic acid (peak 27) was also observed. Hydroxycinnaminic, hydroxybenzoic, and caffeoylquinic acids have been previously reported to be present in *Mentha* species with concentrations frequently below 1%, as observed for the current water-extracted spearmint [31].

Small amounts of flavones, flavonols, and flavanones were detected. Among the flavones, the most representative compound, in terms of quantity, was apigenin (0.19 mg/g) which was identified by comparing the obtained mass spectra with those reported in the literature [26]. Rutin, narirutin, and orientin were recognised using their respective commercial standards, while other compounds, such as luteolin-rutinoside, luteolin-hexoside, and luteolin-glucuronide, were identified by comparison of their relative mass spectra to those reported for other vegetables or natural extracts [20,24]. Rutin, luteolin, and several additional flavones have been reported previously in commercially available spearmint at levels similar to those reported for the current extract. However, the apigenin levels reported for the extract was four-fold greater than that previously reported, although less than 1% in both cases [7].

### 2.2. Characterisation of Volatile Composition

**Figure 2:**
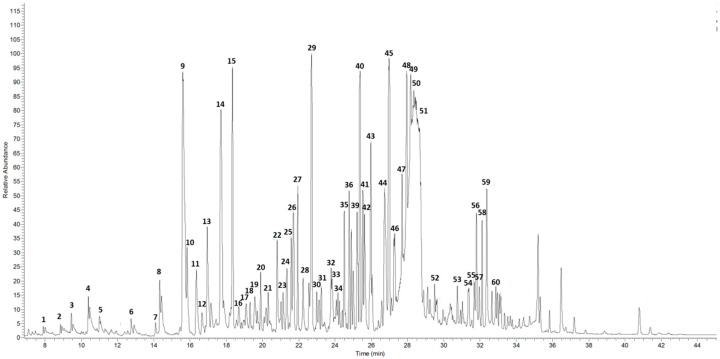
HS-SPME/GC-MS chromatogram of the spearmint extract analyzed. Numbers correspond with the codes indicated at Table 3.

The volatile fraction of dried aqueous spearmint extract was characterised using the HS-SPME/GC-MS technique, which involved obtaining 59 different gas-chromatographic peaks (Figure 2). Peak identification was carried out by comparing recorded mass spectra with those present in the instrument libraries (NIST) and by using the LRI (Linear Retention Index) obtained on two different stationary phase columns (SUPELCOWAX 10 and BP5MS). The detected compounds were semi-quantified using toluene as internal standard (IS). All of the results are listed in Table 3.

Quantitatively, the volatile fraction of the spearmint extract examined had 34.64 ± 10.57 µg/100 mg of volatile compounds. In general, since this extract is water-extracted, the volatile fraction analysis yields percentages of components much lower than those reported in the literature for spearmint leaf material. Ketones were the most representative compounds in this fraction, constituting about 32% of the total volatile amount, followed by terpenoids at 20%. Aldehydes, esters, and furans were also detected at 18%–19% of the total volatile fraction. The highest quantitative individual compounds present in the volatile fraction of the tested spearmint were as follows: *p*-cymene (3.39 ± 0.98 µg/100 mg), isopiperitone and piperitone (2.37 ± 0.94 and 0.69 ± 0.21 µg/100 mg, respectively), dihydroedulan II (two signals: 2.27 ± 0.66 and 0.69 ± 0.09 µg/100 mg), menthone (2.18 ± 0.72 µg/100 mg), *p*-cymen-8-ol (1.96 ± 0.74 µg/100 mg), and β-linalool (1.52 ± 0.43 µg/100 mg). These molecules confer characteristic aromatic notes to the product, such as minty, phenolic, and floral flavours [32].

Traditional mint presents a really distinctive flavour, mostly due to the presence of a particular alcoholic cyclic terpene: menthol. This molecule, besides being well-known as a primary aromatic compound, is used in medicine for gastro-intestinal disorders [44]. In our sample, menthol was not detected. This can be attributed to the fact that the chemical composition of mint leaves, as the composition of essential oil, can be dependent on different agronomical factors as plant maturity, variety, growth region, climatic conditions, and genetics [3]. In contrast, other typical spearmint volatile fraction components, such as menthone, carvone, eugenol, piperitone, and isopiperitone, were detected. These volatiles have been already reported in peppermint and spearmint essential oils as being responsible for the typical mint notes [45,46].

Carvone and piperitone are two oxygenated terpenoids generated during the biosynthesis of terpenes, which starts from geranyl pyrophosphate, and they are derived from d-limonene. In particular, carvone, with its characteristic aromatic note of mint and liquorice, has different applications, such as repellent, medical, and flavour preparation [5]. However, the carvone level recorded in the spearmint extract is 200-fold lower than that previously reported in an aqueous extract of peppermint (~0.2 vs. 40 µg/100 mg extract), another member of the Lamiaceae family [47]. This low carvone level, in agreement with Narasimhamoorthy et al. [11], may cause lesser mint notes in this line relative to native spearmint lines, which could support its palatability in food and beverage applications.

Among ketones, the most abundant were menthone (2.18 ± 0.72 µg/100 mg) and β-damascenone (0.66 ± 0.17 µg/100 mg), which were consistent with results found by Rohloff et al. [46] and Ka et al. [37] for spearmint and peppermint. The spearmint volatile fraction was also rich in alcohols. In addition to the *p*-cymen-8-ol (1.96 ± 0.74 µg/100 mg) as identified in *Mentha* essential oils [4], detectable amounts of 2-acetyl-4-methylphenol, thymol, carvacrol, and *p*-menthen-1-ol were also observed. 

In addition to ketones, terpenoids, and alcohols, several compounds belonging to different chemical classes represented the remaining 18%–19% of the volatile fraction of the dried spearmint powder. Among these minor volatile compounds, dihydroedulan II (two signals: 2.27 ± 0.66 and 0.69 ± 0.09 µg/100 mg) was identified. Dihydroedulan II is a benzopyran compound that has already been detected in the essential oil of *Ocimum basilicum* (basil), another member of the Lamiaceae family [48] but not previously reported in *Mentha spicata*. In accordance with data from Rohloff [46] in peppermint, detectable amounts of *R*-(+)-menthofuran (0.16 ± 0.05 µg/100 mg) were observed. Slight quantities of aldehydes, in particular furfural (0.52 ± 0.12 µg/100 mg) and 5-methyl furfural (0.18 ± 0.03 µg/100 mg), were also detected. Similarly, Ka et al. [37] identified these compounds in distilled extracts from some medicinal plants, such as *Angelica tenuissimae*, pine needles from *Pinus sylvestris*, and leaves of sweet flags (*Acorus gramineus*).

## 3. Materials and Methods

### 3.1. Materials

Methanol, acetonitrile, formic acid, toluene, and C_8_–C_20_ alcane solution were purchased from Sigma-Aldrich (Milan, Italy). Ultrapure water from MilliQ system (Millipore, Bedford, MA, USA) was used throughout the experiment. The proprietary spearmint extract was manufactured by Kemin Foods, L.C. (Des Moines, IA, USA) as described [11,49]. In brief, the spearmint extract was prepared by microwave drying within one hour of harvest followed by extraction of the dried spearmint leaf with acidified water.

### 3.2. Characterization and Quantification of Phenolic Fraction by UHPLC-ESI-MS^n^

The extraction of phenolic compounds was performed on 200 mg of spearmint extract by adding 1 mL of 80% aqueous methanol acidified with formic acid (1%), according to Sánchez-Salcedo et al. (2015) [50]. The solution was shaken in an ultrasonic bath at room temperature for 25 min. The mixture was then centrifuged at 10,480 *g* for 5 min at room temperature. In order to obtain an exhaustive extraction of the phenolic fraction, two additional extractions were performed on the same sample. The three supernatants were pooled before UHPLC-ESI-MS^n^ analyses. Each sample was extracted in quadruplicate.

Methanolic extracts of spearmint were analyzed using an Accela UHPLC 1250 equipped with a linear ion trap-mass spectrometer (MS) (LTQ XL, Thermo Fisher Scientific Inc., San Jose, CA, USA) fitted with a heated-electrospray ionization probe (H-ESI-II; Thermo Fisher Scientific Inc.). Separations were performed using a BlueOrchid C18 column (50 × 2 mm, 1.8 µm particle size, Knauer, Berlin, Germany). The total volume injected was 5 µL and the column oven temperature was 30 °C. Two MS experiments in negative mode were performed according to a previous protocol [51]. Optimal parameters for epicatechin analysis (Experimental Conditions 1) were carried out using the following conditions. The MS was operated using a capillary temperature equal to 275 °C, while the source heater temperature was set to 200 °C. The sheath gas flow was operated at 40 units, while both auxiliary and sweep gas were set to 5 units. The source voltage was 4 kV. The capillary and tube lens voltages were −42 and −118 V, respectively. Elution was performed at a flow rate of 0.3 mL/min. The gradient started with 99% of 0.1% aqueous formic acid, keeping isocratic conditions for 2 min, followed by a 10 min linear gradient of acetonitrile in 0.1% formic acid which started at 1% and was increased to 40%. The acidified acetonitrile was increased to 80% between minutes 12 and 13 min, and maintained for 3 min, followed by 4 min at the starting conditions to re-equilibrate the column. Analyses were carried out using full scan, data-dependent MS^3^ scanning from *m*/*z* 100–1500, with collision-induced dissociation (CID) equal to 30 (arbitrary units). Pure helium gas was used for CID.

The second experimental framework utilized MS with conditions optimized for rosmarinic acid analysis (Experimental Conditions 2). The capillary temperature was set to 275 °C, while the source heater temperature was 50 °C. The sheath gas flow was operated at 40 units, while auxiliary and sweep gas were set to 5 and 0 units, respectively. The source voltage was operated at 4 kV. The capillary and tube lens voltages were −26 and −78 V, respectively. Analyses were carried out using full scan, data-dependent MS^3^ scanning from *m*/*z* 100–1500, with CID equal to 30 (arbitrary units). The chromatographic conditions were identical to those used for the preliminary phenolic analyses.

Quantification was performed using selected ion monitoring mode (SIM) by selecting the relative base peak at the corresponding mass to charge ratio (*m*/*z*) under Experimental Conditions 2, based on rosmarinic acid. Different dilutions of the extract in 0.1% aqueous formic acid (dilution factors ranging from 10–1000) were used to avoid signal saturation and quantify within the linearity range of the reference compounds.

### 3.3. Volatile Extraction and Characterization by Head Space Solid Phase Microextraction (HS-SPME) Coupled with GC-MS Technique

The volatile fraction of the spearmint sample was characterized following the protocol of Cirlini et al. (2012) [34] with slight modifications. Briefly, 100 mg of spearmint extract were placed in a 30 mL glass vial. For each SPME analysis, 100 µL of an aqueous toluene standard solution (348 mg/L) were added to the sample. The vial was stirred in a warm water bath at 35 °C for 45 min. For each sample, a SPME fibre was inserted in the sample head space and the sample was stirred at constant speed. The fibre was then removed and inserted into the GC-MS injector for 2 min for the desorption of the volatiles. The analysis was done in duplicate.

The silica fibre adopted for the analysis was coated with 50/30 µm of divinylbenzene-carboxen-polymethylsiloxane (DVB/Carboxen/PDMS; Supelco, Bellefonte, PA, USA). Before starting the analyses, the fibre was conditioned by inserting it into the GC/MS injector at 230 °C for at least 10 min. All the analyses were performed on a Thermo Scientific Trace 1300 gas-chromatograph coupled to a Thermo Scientific ISQ mass spectrometer equipped with electronic impact (EI) source. The separation of analytes was performed on a SUPELCOWAX 10 capillary column (Supelco, 30 m × 0.25 mm, f.t. 0.25 µm) using helium as carrier gas. The injector temperature was set at 230 °C and splitless mode was used as the injection modality keeping the valve closed for 2 min. The oven temperature started at 50 °C for 3 min and was increased to 200 °C (5 °C/min). The final oven temperature (200 °C) was maintained for 18 min and the auxiliary temperature was set at 230 °C. Full scan mode was chosen as the acquisition mode (*m*/*z* 41–500).

The tentative identification of the volatiles was performed by comparison of the obtained mass spectra with those present in the instrument libraries (NIST). Furthermore, in order to obtain a more confident identification, the linear retention indices (LRI) were calculated on the basis of a C_8_–C_20_ alcane solution analyses. The same procedure was repeated utilizing a different stationary phase column, BP5MS (30 m × 0.25 mm, with 0.25 µm film thickness, SGE Analytical Science, Milan, Italy), on which both the alcane standard solution and spearmint sample were analysed maintaining the same extraction and instrumental conditions as previously described. The semi-quantification of all detected gas-chromatographic signals was performed on the basis of the use of an internal standard (toluene).

## 4. Conclusions

This study reported the comprehensive characterisation of a spearmint extract developed utilizing selective breeding to yield high rosmarinic acid and other phenolic components, with a particular emphasis on the (poly)phenolic and volatile fraction. The use of two different chromatographic techniques, UHPLC, and GC, both coupled to mass spectrometry, allowed for the elucidation of the fingerprint of these two different fractions.

In particular, the use of the UHPLC-ESI-MS^n^ technique allowed us to fully unravel the (poly)phenolic profile of dried spearmint. A total of 66 different molecules were identified on the basis of their characteristic MS^n^ spectra, with 53 of them semi-quantified. The total amount of phenolic compounds was about 260 mg/g extract, which demonstrated that the spearmint extract is a matrix rich in phenolics. The major phenolic compounds in the spearmint extract were represented by rosmarinic acid and its derivatives (88% of the total phenolics). Among the other molecules identified, different salvianolic, caffeoylquinic, hydroxybenzoic, and hydroxycinnamic acids were detected, as well as small amounts of flavones, flavanones, and flavonols. The results of the spearmint extract volatile profile, analysed using the HS-SPME/GC-MS technique, suggested the extract was mainly represented by 59 volatile compounds belonging to different chemical classes, in particular ketones and terpenoids. Attending to the characteristics of plant extracts, the phytochemical composition of this matrix could vary from season to season and even from lot to lot. Regardless of normal variation, these particularly sensitive techniques would allow testing of the authenticity of the product and assist when evaluating its biological and essential properties. On the other hand, the analysis of a higher number of samples, considering factors such as seasonality as well as agricultural practices and crop location would be quite interesting. This fact could be tackled in further studies, although a reductive approach would be needed since it is not feasible to perform this kind of comprehensive identification for large batches of samples.

## Figures and Tables

**Table 1:** Identification of phytochemical compounds by UHPLC-MS^n^ in negative mode under different MS operating conditions.

ID	Compounds	RT (min)	[M − H]^−^ (*m*/*z*)	MS^2^ Ion Fragments (*m*/*z*) ^a^	MS^3^ Ion Fragments (*m*/*z*) ^a^	Exp. 1 ^c^	Exp. 2 ^c^	Identification ^d^
1	Quinic acid	0.57	**191**	**173** ^b^, 111, 127, 85, 93	111, 67	x	x	Std
2	l-malic acid	0.67	**133**	**115**, 87			x	[12]
3	Citric acid	0.77	**191**	**111**, 173	111, 67	x	x	[13]
4	Dihydroxyphenyllactic acid (Danshensu)	2.61	**197**	**179**, 73, 153	135	x		[14]
5	Protocatechuic acid hexoside	2.75	**315**	**153**, 109, 225	109	x		[15]
6	Dihydroxyphenylacetic acid	3.35	**167**	**123**			x	[16]
7	Hydroxybenzoic acid	4.12	**137**	**137**, 93			x	[17]
8	Caftaric acid	4.40	**311**	**149**, 179, 243, 135	103, 87, 131, 59, 149		x	Std
9	Hydroxyphenyllactic acid	4.47	**181**	**163**, 135, 73	119	x	x	[18]
10	Luteolin-8-*C*-glucoside (orientin)	4.83	**447**	357, 327				Std
11	3′-Caffeoylquinic (neochlorogenic acid)	4.96	**353**	**191**, 179, 135, 173	127, 173, 85, 93		x	Std
12	THDBCHMCA ^f^	5.42	**295**	**163**, 113	118		x	[19]
13	Rosmanol	5.44	**345**	**299**	179, 119, 143, 113, 161		x	[20]
14	Coumaric acid	5.52	**163**	**119**		x		[17]
15	Salvianolic acid F	5.56	**313**	**269**, 203, 159	159, 109, 254, 269	x		[14]
16	Dicaffeic acid	5.74	**341**	**281**, 251, 179, 221, 323	179, 221, 135	x	x	[21]
17	5′-Caffeoylquinic (chlorogenic acid)	6.17	**353**	**191**, 179	127, 173, 85, 83		x	Std
18	Caffeic acid	6.25	**179**	**135**	135	x	x	Std
19	Ferulic acid derivative	6.88	**489**	**193**, 235, 295, 265	149, 134, 178	x		Std
20	Rosmarinic acid derivative	6.92	**377**	**359**	161, 179, 197, 223	x	x	Std
21	Rosmarinic acid derivative	7.08	**377**	**359**	161, 179, 197, 223	x	x	Std
22	Feruloylquinic acid	7.15	**367**	**173**, 193, 191	93, 111, 155, 71	x	x	[22]
23	Tetrahydroxy-dimethoxyflavone-hexoside	7.29	**507**	**327**, 345, 477, 489	312, 167, 295	x		[23]
24	Danshensu derivative	7.40	**527**	**197**, 179, 483	179, 73	x		[14]
25	Rosmarinic acid-*O*-caffeic acid	7.61	**539**	**359**, 495, 341, 179	161, 179, 197, 223	x	x	[14]
26	Salvianolic acid J/isomer	7.82	**537**	**339**	229, 295	x	x	[14]
27	Salicylic acid	7.85	**137**	**93**, 137			x	[17]
28	Rosmarinic acid-rutinoside	7.96	**667**	**359**, 487	161, 197, 179, 223	x		Std
29	Quercetin-rutinoside (rutin)	8.07	**609**	**301**, 343, 271, 255, 179	179, 151, 257, 273	x	x	Std
30	Salvianolic acid J/isomer	8.08	**537**	**493**, 295, 339	295, 313, 383	x	x	[14]
31	Luteolin-rutinoside	8.16	**593**	**285**	241, 285, 175, 199, 217	x	x	[24]
32	Rosmarinic acid-*O*-hexoside	8.25	**521**	**359**	161, 197, 179, 223	x	x	Std
33	Luteolin-hexoside	8.26	**447**	**285**	285, 241, 199, 175, 217	x		[24]
34	Luteolin-glucuronide	8.3	**461**	**285**	285, 241	x	x	[20]
35	Salvianolic acid B/E/isomer	8.43	**717**	**519**, 475, 339, 537	475, 339, 365	x	x	[14]
36	Narirutin (Naringenin-7-*O*-rutinoside)	8.45	**625** (579) ^e^	579	271	x	x	Std
37	Salvianolic acid D	8.53	**417**	**373**, 175, 273, 399	175, 197, 223		x	[14]
38	Sagerinic acid	8.66	**719**	**359**, 539, 521, 341	161, 179, 197, 223	x		[16]
39	Salvianolic acid E	8.78	**717**	**519**, 537, 555, 673, 339	339, 321, 295, 229	x	x	[14]
40	Rosmarinic acid	8.86	**359**	**161**, 179, 197, 223	161, 133	x	x	Std
41	Sagerinic acid isomer	8.99	**719**	**359**	161, 179, 197, 223		x	[25]
42	Salvianolic acid A derivative	9.08	**897**	**493**, 295	295, 313, 179		x	Std
43	Lithospermic acid	9.44	**537**	**493**, 359	359, 313, 295	x	x	Std
44	Salvianolic acid B	9.61	**717**	**519**, 321	321, 339, 279, 197, 179	x	x	Std
45	Dehydro-Rosmarinic acid	9.70	**343**	**161**, 179, 135, 223, 197	161, 133	x	x	Std
46	Salvianolic acid B/E/isomer	9.75	**717**	**519,** 357, 555, 673, 321	321, 357, 339	x	x	[14]
47	Rosmarinic acid-dihexoside	9.83	**683**	**521**	359, 161, 197, 223	x		Std
48	G(8-*O*-4)5H	9.88	**373**	**179**, 161, 135, 355, 197	135, 161	x		[14]
49	Salvianolic acid A	10.02	**493**	**295**, 313, 383, 203	159, 277, 109, 267	x	x	Std
50	Acacetin derivative	10.12	**637**	**591**, 283	283, 268	x	x	[18]
51	Salvianolic acid A isomer	10.25	**493**	**295**, 331, 383	159, 277, 109, 267	x	x	[19]
52	Rosmarinic acid derivative	10.70	**551**	**519**, 359, 313	339	x	x	[20]
53	Danshensu derivative	10.87	**689**	**527**, 491	197, 179, 347, 161	x	x	[14]
54	Danshensu derivative	10.90	**691**	**529**, 493, 511	197, 179, 349, 151	x	x	[14]
55	Danshensu derivative	11.07	**689**	**527**	197, 179, 347	x	x	[14]
56	Rosmarinic acid derivative	11.07	**691**	**359**, 511, 341, 529	161, 179, 197, 223	x		Std
57	Apigenin	11.17	**269**	**269**, 225, 149, 241	181, 197, 225, 183	x		[26]
58	Salvianolic acid A isomer	11.22	**493**	**359**, 357, 313	161, 179, 197, 223	x	x	[19]
59	Cyclolariciresinol	11.26	**359**	**345**, 161	329, 326	x	x	[27]
60	Salvianolic acid B derivative	11.40	**879**	**519**, 699, 339	339	x		[25]
61	Rosmarinic acid derivative	12.33	**571**	**525**	341, 359, 161, 179, 221		x	Std
62	Rosmarinic acid derivative	12.69	**525**	**359**, 341, 161, 179	161, 179, 197, 223	x		Std
63	Rosmarinic acid derivative	13.04	**507**	**359**, 341, 179	161, 179, 197, 223	x	x	Std
64	Rosmarinic acid derivative	13.24	**849**	**359**, 687, 669	161, 179, 197, 223	x	x	Std
65	Acacetin	13.54	**283**	**268**, 269	268, 269, 240	x	x	[18]
66	Rosmarinic acid derivative	13.82	**507**	**359**, 341, 179	161, 179, 197, 223	x	x	Std

^a^ Fragment ions are listed in order of relative abundances; ^b^ MS^2^ ions in bold were those subjected to MS^3^ fragmentation; ^c^ Exp. 1, detected under experimental condition 1 (epicatechin); Exp. 2, experimental condition 2 (rosmarinic acid); ^d^ Identification means identification mode: [Reference number] or Std (compound identified by comparing retention times and MS data with those of reference compounds). Some compounds have been considered “derivatives” since parts of their spectra match those of their corresponding parent compounds but they cannot be fully identified; ^e^ The molecular ion is a formic acid adduct (+46); ^f^ THDBCHMCA: 1,2,6,7-tetrahydroxy-5*H*-dibenzo[*a*,*d*]cycloheptene-5-methyl-11-carboxylic acid.

**Table 2:** Quantitative results (mg/g sample) for polyphenolic fraction of the spearmint extract analyzed.

ID ^a^	Compounds	Quantified as…	Concentration (mg/g)
4	Dihydroxyphenyllactic acid (Danshensu)	Caffeic acid	0.77 ± 0.09
5	Protocatecuic acid hexoside	Caffeic acid	0.04 ± 0.00
7	Hydroxybenzoic acid	Caffeic acid	0.57 ± 0.07
8	Caftaric Acid	Caftaric acid	2.18 ± 0.30
9	Hydroxyphenyllactic acid	Caffeic acid	0.07 ± 0.00
10	Luteolin-8-*C*-glucoside (orientin)	Luteolin-4-glucoside	0.02 ± 0.00
11	3′-Caffeoylquinic (neochlorogenic acid)	3′-Caffeoylquinic ^b^	1.79 ± 0.22
14	Coumaric acid	Caffeic acid	0.03 ± 0.00
15	Salvianolic Acid F	Caffeic acid	0.01 ± 0.00
16	Dicaffeic acid	Caffeic acid	0.09 ± 0.00
17	5′-Caffeoylquinic (chlorogenic acid)	5′-Caffeoylquinic ^b^	1.16 ± 0.08
18	Caffeic acid	Caffeic acid	0.71 ± 0.06
20	Rosmarinic acid derivative	Rosmarinic acid	2.17 ± 0.25
21	Rosmarinic acid derivative	Rosmarinic acid	1.61 ± 0.11
22	Feruloylquinic acid	3′-Caffeoylquinic	0.11 ± 0.00
24	Danshensu derivative	Caffeic acid	0.01 ± 0.00
25	Rosmarinic acid-*O*-caffeic acid	Rosmarinic acid	0.05 ± 0.00
26	Salvianolic acid J/isomer	Salvianolic acid A	1.84 ± 0.17
28	Rosmarinic acid-rutinoside	Rosmarinic acid	0.17 ± 0.00
29	Quercetin-rutinoside (rutin)	Rutin ^b^	0.01 ± 0.00
30	Salvianolic acid J/isomer	Salvianolic acid A	0.36 ± 0.05
31	Luteolin-rutinoside	Luteolin-4-glucoside	0.17 ± 0.01
32	Rosmarinic acid-*O*-hexoside	Rosmarinic acid	0.28 ± 0.03
33	Luteolin-hexoside	Luteolin-4-glucoside	0.02 ± 0.00
34	Luteolin-7-glucuronide	Luteolin-4-glucoside	0.13 ± 0.00
35	Salvianolic acid B/E/isomer	Salvianolic acid B	0.41 ± 0.05
36	Narirutin (Naringenin-7-*O*-rutinoside)	Narirutin ^b^	0.04 ± 0.01
37	Salvianolic Acid D	Rosmarinic acid	0.29 ± 0.02
38	Sagerinic Acid	Rosmarinic acid	8.93 ± 1.10
39	Salvianolic Acid E	Salvianolic acid B	0.16 ± 0.02
40	Rosmarinic Acid	Rosmarinic acid ^b^	173.76 ± 11.52
41	Sagerinic Acid isomer	Rosmarinic acid	40.05 ± 2.20
42	Salvianolic Acid A derivative	Salvianolic acid A	1.44 ± 0.30
43	Lithospermic Acid	Lithospermic acid ^b^	3.81 ± 0.26
44	Salvianolic Acid B	Salvianolic acid B ^b^	1.35 ± 0.16
45	Dehydro-Rosmarinic Acid	Rosmarinic acid	0.52 ± 0.01
46	Salvianolic acid B/E/isomer	Salvianolic acid B	0.30 ± 0.03
47	Rosmarinic acid-dihexoside	Rosmarinic acid	0.16 ± 0.01
49	Salvianolic Acid A	Salvianolic acid A ^b^	7.79 ± 0.52
51	Salvianolic Acid A isomer	Salvianolic acid A	0.31 ± 0.06
52	Rosmarinic acid derivative	Rosmarinic acid	0.28 ± 0.02
53	Danshensu derivative	Caffeic acid	0.06 ± 0.00
54	Danshensu derivative	Caffeic acid	0.03 ± 0.00
55	Danshensu derivative	Caffeic acid	0.05 ± 0.00
56	Rosmarinic acid derivative	Rosmarinic acid	0.10 ± 0.01
57	Apigenin	Daidzein	0.19 ± 0.01
58	Salvianolic Acid A isomer	Salvianolic acid A	0.69 ± 0.02
60	Salvianolic Acid B derivative	Salvianolic acid B	0.05 ± 0.00
61	Rosmarinic acid derivative	Rosmarinic acid	0.67 ± 0.04
62	Rosmarinic acid derivative	Rosmarinic acid	0.09 ± 0.00
63	Rosmarinic acid derivative	Rosmarinic acid	0.01 ± 0.00
64	Rosmarinic acid derivative	Rosmarinic acid	1.30 ± 0.16
66	Rosmarinic acid derivative	Rosmarinic acid	0.09 ± 0.00
		*Hydroxybenzoic acids* ^c^	0.61 ± 0.08
		*Hydroxycinnamic acids*	3.00 ± 0.36
		*Caffeoylquinic acids*	3.06 ± 0.27
		*Hydroxyphenylpropanoic acids*	0.99 ± 0.10
		*Rosmarinic acid derivatives*	230.50 ± 13.5
		*Salvianolic acids*	14.70 ± 1.19
		*Flavones*	0.53 ± 0.02
		*Flavonols*	0.01 ± 0.00
		*Flavanones*	0.04 ± 0.01
		*Total Phenolics*	262.97 ± 15.90

^a^ See Table 1 for peak assignment; ^b^ Quantified by comparison with its corresponding standard; ^c^ hydroxybenzoic acids include compound **5** and **7**; hydroxycinnamic acids, compounds **8**, **14**, **16**, and **18**; caffeoylquinic acids, **11**, **17**, and **22**; hydroxyphenylpropanoic acids, **4, 9, 24**, and **53**–**55**; rosmarinic acid derivatives, **20**, **21**, **25**, **28**, **32**, **37**, **38**, **40**, **41**, **45**, **47**, **52**, **56**, **61**–**64**, and **66**; salvianolic acids, **15**, **26**, **30**, **35**, **39**, **42**, **44**, **46**, **49**, **51**, **58**, and **60**; flavones, **31**, **33**, **34**, and **57**; flavonols, **29**; and flavanones, **36**. Mean (*n* = 3) ± SD.

**Table 3:** Identification of volatile compounds from the spearmint extract, with relative aromatic notes, calculated LRIs, identification methods, references, and relative amounts.

ID	Identification	Flavour Note [32]	LRI-Wax	LRI-BP5 ^a^	Identification Method	Ref.	Concentration (µg/100 mg)
1	Ethylbenzene	Prunus	**1127**	**871**	MS + LRI	[33]	0.04 ± 0.01
2	d-Limonene	Sweet, citrus and peely	**1200**	**1024**	MS + LRI	[34]	0.04 ± 0.01
3	Cosmene	Dahlia, Laurus nobilis	**1219**	**1006**	MS + LRI	NIST	0.24 ± 0.08
4	Cosmene (isomer)		**1252**	**1142**	MS + LRI	NIST	0.41 ± 0.03
5	o-cymene	Lavander and cypress oil	**1274**	**1022**	MS + LRI	[35]	0.06 ± 0.01
6	Methyl-heptenone	Fruity, apple, musty, ketonic and creamy	**1343**		MS		0.05 ± 0.01
7	(*z*)-3-hexen-1-ol	Green, grassy, melon rind-like	**1387**	**853**	MS + LRI	[36]	0.07 ± 0.01
8	Amyl ethyl carbinol	Earthy	**1395**	**996**	MS		0.29 ± 0.09
9	*p-*cymenene	Phenolic	**1444**	**1090**	MS + LRI	[35]	3.39 ± 0.98
10	Amyl vinyl carbinol	Earthy	**1453**	**979**	MS + LRI	[34]	0.46 ± 0.11
11	Furfural	Bready	**1473**	**828**	MS + LRI	[20]	0.52 ± 0.12
12	α-ionene	Plum	**1485**		MS		0.13 ± 0.01
13	Dihydroedulan II	(not reported)	**1496**	**1292**	MS + LRI	[37]	0.69 ± 0.09
14	Dihydroedulan II	(not reported)	**1526**	**1297**	MS + LRI	[37]	2.27 ± 0.66
15	β-linalool	Floral	**1551**	**1099**	MS + LRI	[38]	1.52 ± 0.43
16	(*R*)-(+)-menthofuran	Minty	**1565**	**1159**	MS + LRI	[39]	0.16 ± 0.05
17	5-methylfurfural	Caramellic	**1582**	**957**	MS + LRI	[38]	0.18 ± 0.03
18	α-ionone	Floral	**1590**	**1428**	MS + LRI	[33]	0.14 ± 0.02
19	(not identified)		**1602**				0.27 ± 0.08
20	Hotrienol	Sweet tropical	**1615**	**1105**	MS + LRI	[40]	0.38 ± 0.19
21	trans-p-metha-2,8-dienol	Minty	**1632**	**1121**	MS + LRI	[35]	0.12 ± 0.03
22	Safranal	Woody, spicy, phenolic, camphoreous	**1653**	**1196**	MS		0.53 ± 0.13
23	3-furanmethanol	Tobacco	**1667**	**851**	MS + LRI	[41]	0.18 ± 0.01
24	Tetramethyl-indane	(not reported)	**1676**		MS		0.42 ± 0.09
25	(not identified)		**1686**				0.33 ± 0.04
26	Ethyl cyclopentenolone	Caramellic	**1691**	**1087**	MS		0.75 ± 0.18
27	p-menthen-1-ol	Floral, minty, eucalyptus	**1701**		MS		0.65 ± 0.19
28	4,7-dibenzofuran	(not reported)	**1714**		MS		0.33 ± 0.06
29	Menthone	Mentholic	**1735**	**1148**	MS + LRI	[35]	2.18 ± 0.72
30	Camphor	Camphoreous	**1748**	**1145**	MS + LRI	[35]	0.20 ± 0.02
31	2-piperidin methenamine	(not reported)	**1759**		MS		0.19 ± 0.08
32	1-(1-butenyl)pyrrolidine	(not reported)	**1783**		MS		0.17 ± 0.05
33	Methyl salicylate	Minty	**1785**	**1205**	MS + LRI	[33]	0.21 ± 0.13
34	trans-geraniol	Floral	**1804**	**1377**	MS + LRI	NIST	0.10 ± 0.03
35	Teresantalol	Magnolia	**1816**	**1205**	MS		0.52 ± 0.12
36	β-damascenone	Woody, sweet, fruity, earthy	**1828**	**1381**	MS + LRI	[38]	0.66 ± 0.17
37	5-isoproprenyl-2-methylcyclopent-1-enecarboxaldehyde	(not reported)	**1834**		MS		0.43 ± 0.08
38	Calamenene	Herbal	**1839**	**1525**	MS + LRI	[33]	0.34 ± 0.11
39	Piperitenone	Herbal, minty	**1849**	**1268**	MS + LRI	[35]	0.69 ± 0.21
40	*p*-cymen-8-ol	Sweet, fruity, cherry, coumarin	**1857**	**1175**	MS + LRI	[33]	1.96 ± 0.74
41	Exo-2-hydroxy cineole	Eucalyptus, basilicum	**1864**		MS		0.36 ± 0.01
42	3,6-dimethyl-phenyl-1,4-diol	(not reported)	**1868**		MS		0.44 ± 0.02
43	Longipinene	Hinoki, cypress	**1884**	**1350**	MS + LRI	[42]	0.74 ± 0.01
44	Isopiperitenone	Minty	**1932**	**1340**	MS + LRI	NIST	2.37 ± 0.94
45	Damascenone (isomer)		**1948**		MS		0.56 ± 0.12
46	Mint lactone	Sweet, creamy, coumarinic and coconut	**1967**		MS		0.46 ± 0.03
47	α,β-dihydro-β-ionone	Woody	**1979**	**1406**	MS		1.17 ± 0.69
48	Seudenone	Nutty	**1990**	**1050**	MS + LRI	NIST	0.50 ± 0.19
49	Dihydroxy-durene	(not reported)	**1998**	**1322**	MS		0.31 ± 0.23
50	Cinerolon	Myrthus	**2011**	**1403**	MS		0.64 ± 0.43
51	Carvone	Minty, licorice	**2054**	**1239**	MS + LRI	[33]	0.18 ± 0.07
52	1-acetoxy-*p*-menth-3-one	Minty	**2114**		MS		0.16 ± 0.05
53	2,6-diisopropyl naphtalene	(not reported)	**2144**		MS		0.33 ± 0.08
54	(naphtalene derivative)		**2158**		MS		0.15 ± 0.05
55	Eugenol	Spicy	**2164**	**1354**	MS + LRI	[35]	0.75 ± 0.44
56	4-ethylphenol	Phenolic	**2171**	**1175**	MS + LRI	[38]	0.17 ± 0.01
57	Thymol	Herbal	**2179**	**1289**	MS + LRI	[35]	0.62 ± 0.29
58	2-acetyl-4-methylphenol	Sweet heavy floral herbal	**2190**	**1180**		[43]	0.95 ± 0.41
59	Carvacrol	Spicy	**2204**	**1298**	MS + LRI	[35]	0.12 ± 0.03

^a^ No value means not found in literature. Mean (*n* = 2) ± SD.

## Abbreviations

The following abbreviations are used in this manuscript:

CID	collision-induced dissociation
GC-MS	gas chromatography-mass spectrometry
LIR	linear retention indices
HS-SPME	head space solid-phase microextraction
UHPLC-ESI-MS^n^	ultra-high performance liquid chromatography-electrospray ionization-mass spectrometry
